# A Clinical Suspicion That Led to the Diagnosis of May-Thurner Syndrome

**DOI:** 10.7759/cureus.76463

**Published:** 2024-12-27

**Authors:** Ana Raquel Gaspar

**Affiliations:** 1 Family Medicine, USF Progresso e Saúde - Tocha, Cantanhede, PRT

**Keywords:** family medicine, may-thurner syndrome, re-vascularization, unilateral leg edema, vascular anomaly

## Abstract

May-Thurner syndrome is an anatomical anomaly characterized by venous compression of the iliac vein by the arterial system. It is more common in women. It may be asymptomatic or lead to symptoms related to hypertension/venous occlusion, namely, edema of the lower limb. The exact value of its prevalence is unknown.

This case reports a 52-year-old woman with a personal history of cervical cancer. She went to her family doctor in June 2021 with complaints of edema of the left thigh, evolving over one week, without associated trauma or pain. She was directed to the emergency service, where after carrying out blood analysis, an indication for topical anti-inflammatories and monitoring of alarm signs was given. She went to the emergency service two more times in the next month with the same indications for treatment.

In September 2022, during a family doctor's appointment, she again mentioned concerns regarding asymmetry of her legs. On objective examination, varicose veins were observed bilaterally. Blood analysis and arterial and venous echo-Doppler were required. There were no changes in the blood analysis. The echo-Doppler was not very conclusive, showing only slightly insufficient perforating veins. It was decided to request an abdominopelvic computed tomography (CT) whose result in February 2023 demonstrated extrinsic compression at the emergence of the left common iliac vein by the right iliac artery, likely related to May-Thurner syndrome.

This clinical case recalls the importance of the family doctor in the longitudinal monitoring of his patients. The unique opportunity to learn about their background and evaluate them when surveying diagnostic hypotheses led to an unexpected diagnosis.

## Introduction

May-Thurner syndrome is an anatomical anomaly characterized by venous compression of the iliac vein, most frequently the left common iliac, by the arterial system. One of the many postulated mechanisms that cause May-Thurner syndrome explains that this compression can be responsible for a continuous trauma of the venous system by the arterial pulsations conditioning inflammation of the vein and the development of scar injuries, leading to clot formation. This process can generate complications such as chronic venous insufficiency due to valve incompetence, deep vein thrombosis, lymphedema, and stasis ulcers [1]. It is more common in women between the third and fifth decades of life [2]. It may be asymptomatic or lead to symptoms related to hypertension/venous occlusion, namely, edema of the lower limb. Because it can be asymptomatic, the exact value of its prevalence is unknown [3,4]. It is frequently misdiagnosed. 

## Case presentation

A 52-year-old woman, belonging to a nuclear family, was in phase VI of the Duvall cycle. Personal history of smoking (20 smoking pack years), asthma, depressive disorder, chronic low back pain, and cervical cancer (underwent hysterectomy and anixectomy in 2002). She was medicated with an inhaler and an anti-inflammatory for pain crises. There were no known drug allergies.

**Figure 1:**
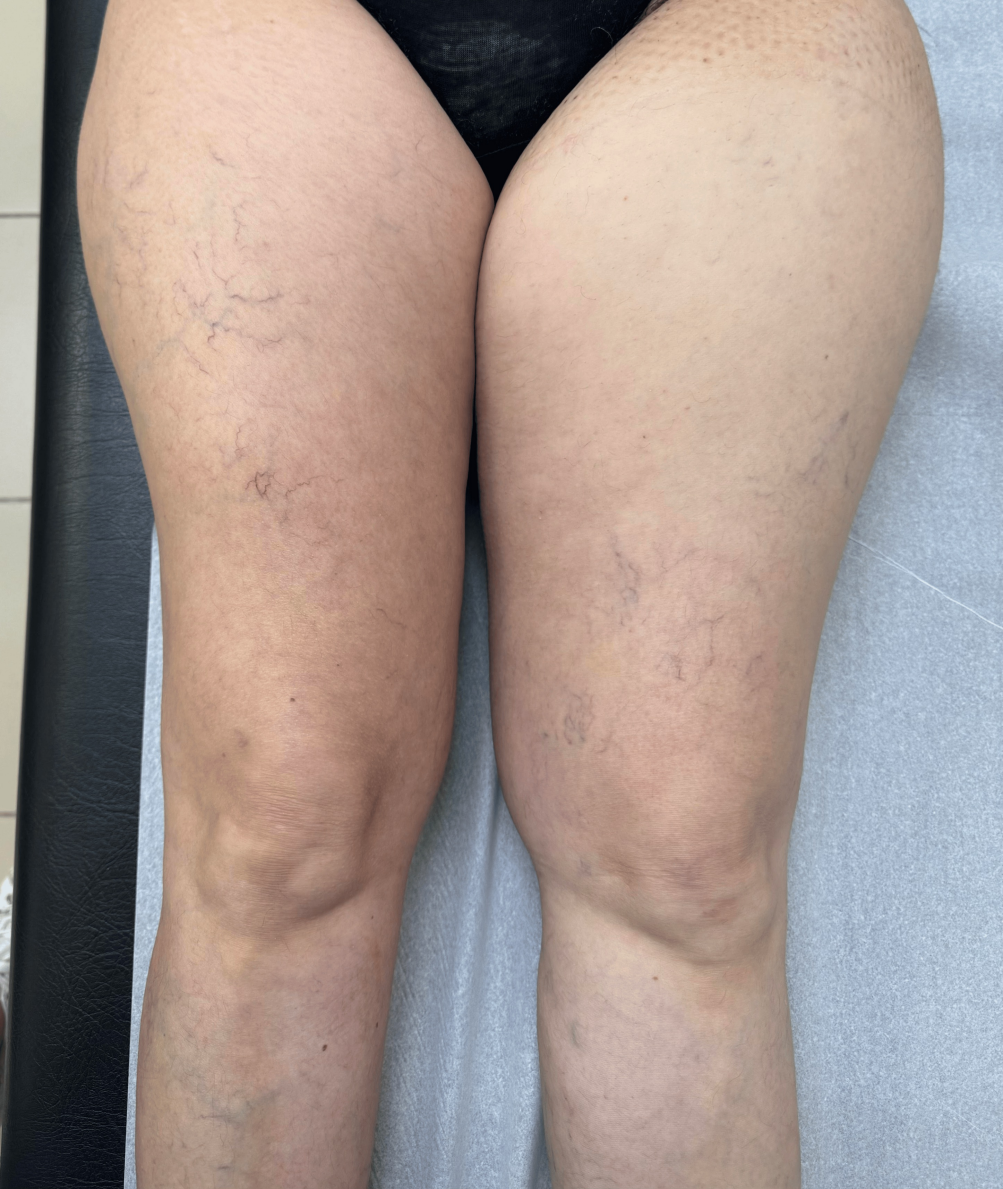
Patient presenting edema of the left thigh

She went to the family doctor in June 2021 with complaints of edema of the left thigh, with one week of evolution, without associated trauma and pain. On objective examination, asymmetry in the diameter of the thighs was confirmed (Figure 1). She presented palpable pulses, without venous cords or inflammatory signs. She was referred to the emergency service, where after carrying out a blood analysis, an indication for topical anti-inflammatory and monitoring of alarm signs was given.

Due to the persistence of symptoms, she went to the emergency service twice more in July 2021. In both visits, edema of the left leg with discrete inflammatory signs (redness and heat)was noticed. Another blood analysis was performed, with no changes, and ultrasounds of the thigh were performed, with no signs of deep vein thrombosis and with normal permeability of the venous system. She was discharged with indications of analgesia, rest, and lifting of the leg. 

**Figure 2:**
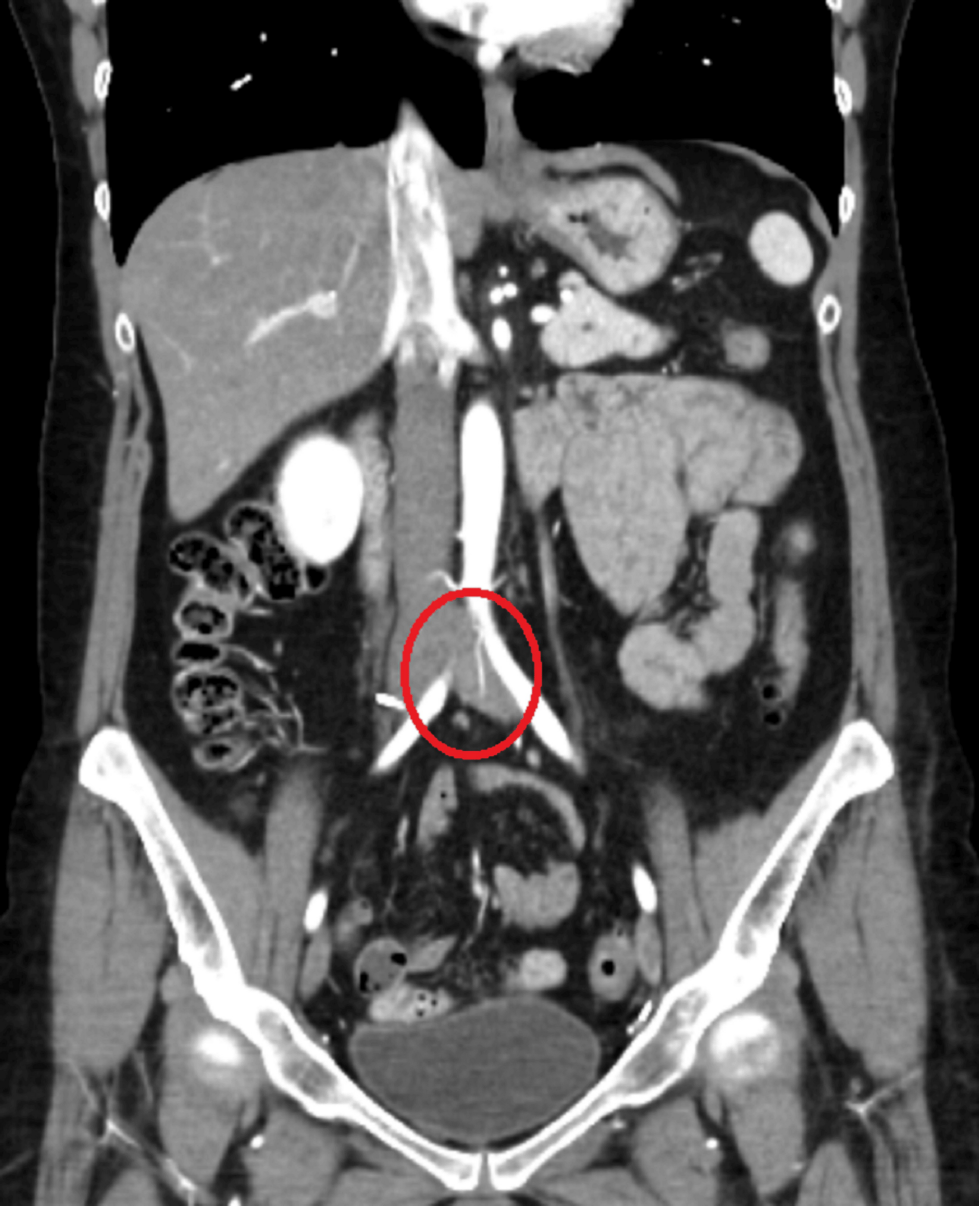
Extrinsic compression of the emergence of the left common iliac vein by the right iliac artery

In September 2022, during a family doctor's appointment, she mentioned again the concerns regarding the asymmetry of the left lower limb. On objective examination, varicose veins were observed bilaterally, with the rest of the examination overlapping. Blood analysis and arterial and venous echo-Doppler were required. In October, she returned with the results of the exams: the blood analysis without changes and echo-Doppler were not very conclusive, showing only slightly insufficient perforating veins. It was decided to request an abdominopelvic CT scan. Due to the personal history of cervical carcinoma, it was thought that a potential recurrence of the cervical cancer or its metastasis could be causing a venous obstruction that explained the symptoms. The result in February 2023 demonstrated extrinsic compression of the emergence of the left common iliac vein by the right iliac artery, with a reduction of the lumen by more than 50%, likely related to May-Thurner syndrome (Figure 2). These CT findings were crucial for the diagnosis. She was next guided for a vascular surgery consultation where, after confirming the diagnosis, surgical treatment with a venous stent was proposed.

## Discussion

May-Thurner syndrome is an underdiagnosed cause of iliofemoral deep vein thrombosis [5], accounting for 2% to 5% of all deep vein thromboses [6]. Iliofemoral venous thromboses can be extensive [7] and, without proper diagnosis and treatment, have significant morbidity [8].

The diagnosis of May-Thurner syndrome requires a comprehensive physical examination and imaging [9]. The physical examination should include a complete set of vital signs, a cardiopulmonary examination, and an evaluation of both lower extremities [6]. A lower extremity duplex ultrasound may fail to examine the iliac vein segments, which means that May-Thurner syndrome is frequently missed in these symptomatic patients. A CT scan is a useful modality [10].

The treatment goals in symptomatic May-Thurner syndrome are to reestablish venous flow [11], alleviate strictures and venous hypertension, and reduce the incidence of postthrombotic syndrome [6].

We should highlight that the Iliac vein compression may be due to underlying malignancy, lymphadenopathy, hematoma, or cellulitis [6,12]. Anatomic variants or disease processes that may compress the iliac vein include uterine leiomyoma, aortoiliac aneurysm, retroperitoneal fibrosis, and osteophyte [6].

In emergency care, the correct complementary diagnostic exams are not always available or are not requested because of the failure to recognize conditions like the May-Thurner syndrome. In this clinical case, the patient only received her diagnosis due to her persistent demands for health care.

Through a collaboration of the family doctor, the radiologist, and vascular surgeons, the diagnosis was made, and it was crucial for choosing treatment and avoiding future complications. The surgery of the patient went well and nowadays, she has symmetrical lower limbs.

## Conclusions

This clinical case shows the importance of the family doctor in the longitudinal monitoring of their patients. The unique opportunity to learn about their background and evaluate them when surveying diagnostic hypotheses led to a diagnosis that, although unexpected, gave name to a complaint that had been evolving for more than one year. It is also important to highlight the value of multidisciplinary collaboration between different medical specialties in follow-up and treatment.

We must always value the complaints of our patients and look for causes of persistent symptoms. A timely diagnosis reduces unnecessary visits to the emergency department and improves accessibility to healthcare.
